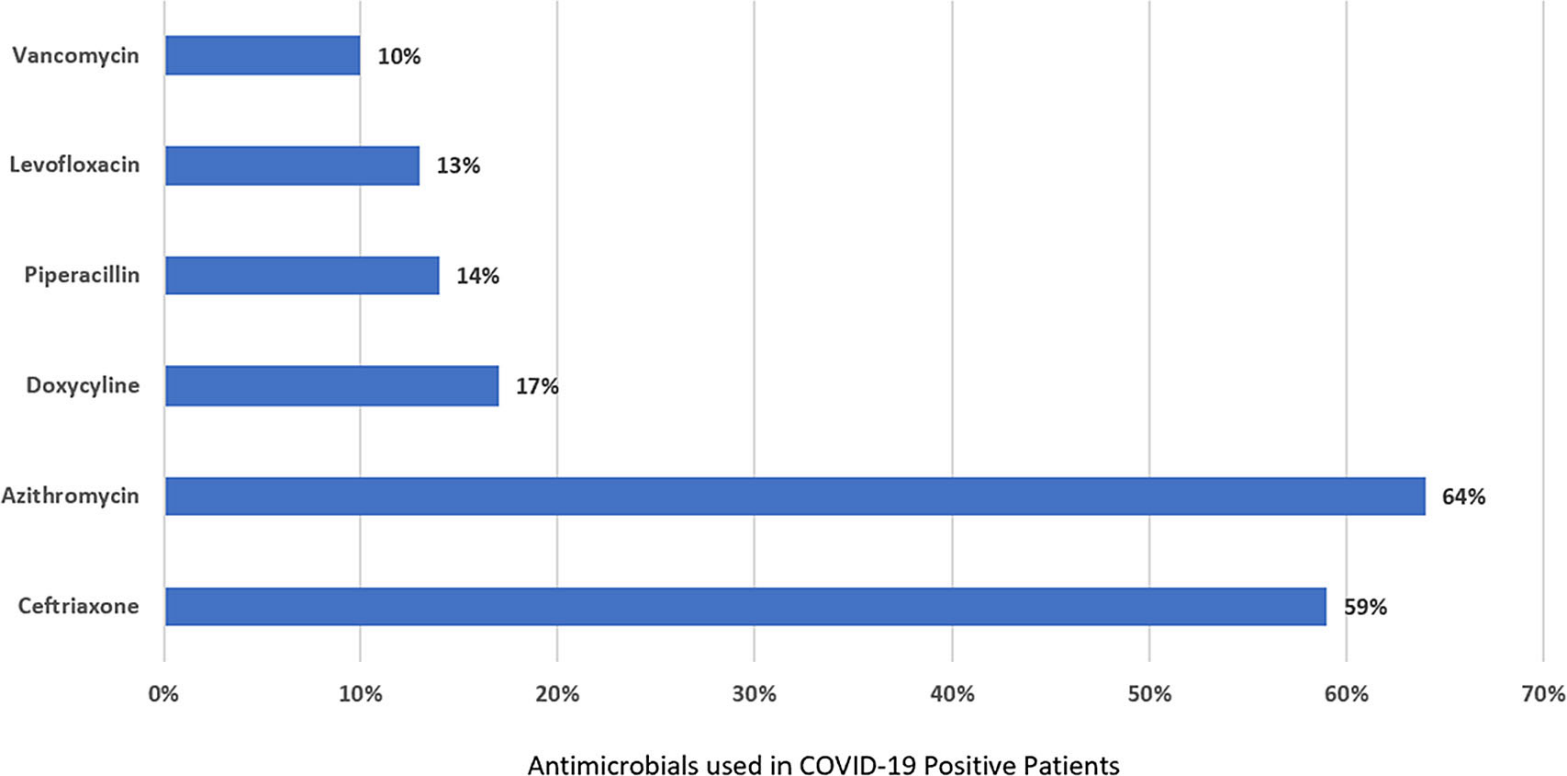# Selection and Duration of Therapy as a Target for Antimicrobial Stewardship during COVID-19: Levees for a Hundred-Year Flood

**DOI:** 10.1017/ash.2021.31

**Published:** 2021-07-29

**Authors:** Alfredo Mena Lora, Rodrigo Burgos, Ella Li, Nichelle Simpkins, Fischer Herald, John Marchionne, Mirza Ali, Eden Takhsh, Sherrie Spencer, Candice Krill, Susan Bleasdale, Scott Borgetti

## Abstract

**Background:** The disease caused by SARS-CoV-2, COVID-19, has caused a pandemic leading to strained healthcare systems worldwide and an unprecedented public health crisis. The hallmark of severe COVID-19 is lower respiratory tract infection (LRTI) and hypoxia requiring hospitalization. A paucity of data on bacterial coinfection and a lack of therapeutic options for COVID-19 during the first surge of cases has increased pressure on antimicrobial use and has challenged antimicrobial stewardship programs (ASPs). We implemented a multimodal approach to antimicrobial stewardship in an urban safety-net community hospital targeting selection and duration of therapy. **Methods:** Retrospective review of cases during the first wave of COVID-19 in a 151-bed urban safety-net community hospital from March to June 2020. EMR order sets (Figure 1) and prospective audit and feedback by ASPs targeting empiric antimicrobial selection and duration were implemented as part of the COVID-19 response. Hospitalized patients with COVID-19 were reviewed retrospectively. Demographic information was collected. Data on antimicrobial use were tabulated, including selection and duration of antimicrobials (Figure 1). **Results:** In total, 302 patients were reviewed, of whom 221 (73%) received empiric antimicrobials. The most commonly used antimicrobials were ceftriaxone and azithromycin (Figure 2). Days of therapy per 1,000 patient days (DOT/1,000 PD) for ceftriaxone increased from 71 in the quarter prior to 113 during the study period. Average duration of therapy was 6 days. In the ICU, average duration was 8 days compared to 5 days in non-ICU settings. Average durations of parenteral therapy were 5.54 days in the ICU and 3.36 days in non-ICU settings. Procalcitonin was obtained in 37 cases (17%) and ranged from 0.09 to 12.57 ng/mL, with an average of 1.21 ng/mL. No cases had documented bacterial coinfection (Figure 1). **Conclusions:** Antimicrobials were commonly prescribed during the first wave of COVID-19 in a safety-net community hospital. Procalcitonin did not guide therapy nor did lack of documented coinfection change physician behavior. With limited resources, ASP successfully guided clinicians toward IDSA guideline recommendations for selection and duration, as evidenced by antimicrobial use. During this unprecedented surge of LRTIs, a multimodal approach to antimicrobial stewardship was used and guided toward early transition to oral agents and shorter durations.

**Funding:** No

**Disclosures:** None

**Figure 1.**
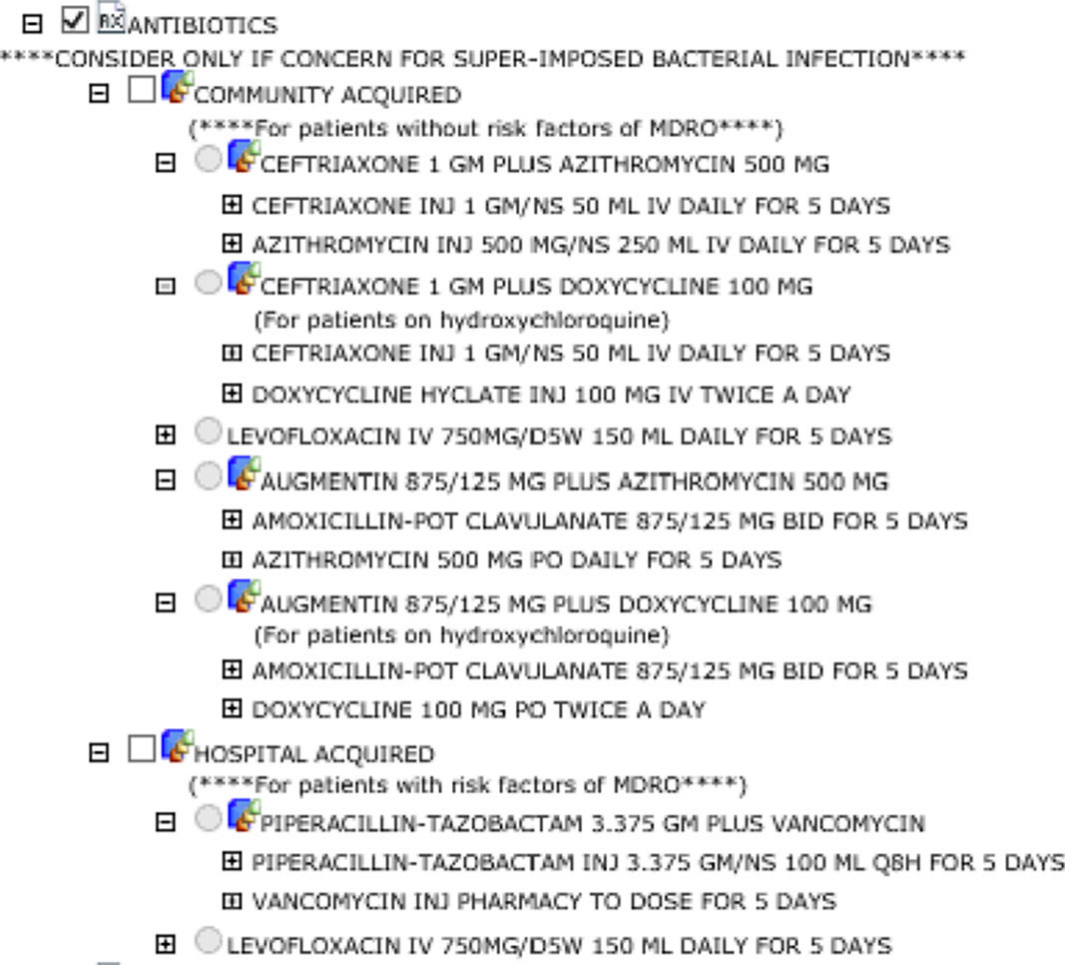

**Figure 2.**